# Minimized Computations of Deep Learning Technique for Early Diagnosis of Diabetic Retinopathy Using IoT-Based Medical Devices

**DOI:** 10.1155/2022/7040141

**Published:** 2022-09-14

**Authors:** Shahnawaz Ayoub, Mohiuddin Ali Khan, Vaishali Prashant Jadhav, Harishchander Anandaram, T. Ch. Anil Kumar, Faheem Ahmad Reegu, Deepak Motwani, Ashok Kumar Shrivastava, Roviel Berhane

**Affiliations:** ^1^Department of Computer Science and Engineering, Shri Venkateshwara University, NH-24, Venkateshwara Nagar, Rajabpur, Gajraula, Dist: Amroha, Uttar Pradesh, India; ^2^Department of Computer & Network Engineering, College of Computer Science and Information Technology, Jazan University, Jazan, Saudi Arabia; ^3^St. Francis Institute of Technology, Borivali, Mumbai 103, Maharashtra, India; ^4^Centre for Excellence in Computational Engineering and Networking, Amrita Vishwa Vidyapeetham, Coimbatore, Tamil Nadu, India; ^5^Department of Mechanical Engineering, Vignan's Foundation for Science Technology and Research, Vadlamudi, Guntur Dt., Andhra Pradesh, India; ^6^Department of Computer Science and Information Technology, Jazan University, Saudi Arabia; ^7^Department of Computer Science and Engineering, Amity University, Gwalior, Madhya Pradesh, India; ^8^Department of Chemical Engineering, College of Biological and Chemical Engineering, Addis Ababa Science and Technology University, Addis Ababa, Ethiopia

## Abstract

Diabetes mellitus is the main cause of diabetic retinopathy, the most common cause of blindness worldwide. In order to slow down or prevent vision loss and degeneration, early detection and treatment are essential. For the purpose of detecting and classifying diabetic retinopathy on fundus retina images, numerous artificial intelligence-based algorithms have been put forth by the scientific community. Due to its real-time relevance to everyone's lives, smart healthcare is attracting a lot of interest. With the convergence of IoT, this attention has increased. The leading cause of blindness among persons in their working years is diabetic eye disease. Millions of people live in the most populous Asian nations, including China and India, and the number of diabetics among them is on the rise. To provide medical screening and diagnosis for this rising population of diabetes patients, skilled clinicians faced significant challenges. Our objective is to use deep learning techniques to automatically detect blind spots in eyes and determine how serious they may be. We suggest an enhanced convolutional neural network (ECNN) utilizing a genetic algorithm in this paper. The ECNN technique's accuracy results are compared to those of existing approaches like the K-nearest neighbor approach, convolutional neural network, and support vector machine with the genetic algorithm.

## 1. Introduction

With 463 million cases worldwide and a projected 700 million cases by 2045 [1], diabetes mellitus is a severe public health issue. The most prevalent eye condition associated with diabetes, diabetic retinopathy (DR), affects at least one-third of diabetics [2]. Any diabetic patient, regardless of how severe their condition is, can develop DR, which is defined by increasing vascular disturbances in the retina brought on by persistent hyperglycemia [3]. Approximately 93 million people worldwide are thought to have DR, making it the largest cause of blindness among working-age adults [4]. The community has begun to pay attention to the confluence of AI and IoT for effective smart healthcare systems in recent years. This convergence makes the detection of many illnesses more effective than ever. One of the most common chronic diseases in the world, diabetes develops when the body is unable to properly use or manufacture the hormone insulin. According to the World Health Organization (WHO), diabetes was a factor in more than 1.6 million fatalities in 2016 [5–10]. Blood glucose levels in diabetic people frequently create high levels, which can harm and fail the body's organs. The International Diabetes Federation (IDF) reports that 1 in 10 persons have diabetes, which is a significant issue. Diabetes complications could lead to heart attacks, kidney failure, severe eyesight difficulties, etc. Although the electroretinography (ERG), retinal blood flow, and retinal blood vessel calibre may all play a role in the initial diagnosis of DR [11], fundus examination is the mainstay of early diagnosis in clinical settings [12]. One of the most popular ways to determine the degree of DR is by the use of fundus photography, a quick, noninvasive, well-tolerated, and widely accessible imaging technique [13]. Ophthalmologists use fundus pictures to view retina lesions at high resolution in order to diagnose and grade the severity of diabetic retinopathy. However, manually diagnosing DR from fundus images requires a high level of expertise and effort from a qualified ophthalmologist, particularly in densely populated or remote areas like in India and Africa, where the number of people with diabetes and DR is projected to increase dramatically in the coming years, while the number of ophthalmologists is disproportionally low [14–17]. The scientific community has been inspired to create computer-aided diagnosis methods in order to lessen the expense, time, and effort required for a medical expert to diagnose DR. Deep Learning (DL) applications for precise DR detection and categorization are now possible thanks to recent developments in artificial intelligence (AI) and the expansion of computer resources and capabilities. This review paper presents and critically analyzes new DL-based approaches for DR detection and classification that were published after 2016. Although numerous review publications on the use of deep learning techniques on DR have been published in recent years [18–29], the majority of them only cover particular facets of the pipeline for data processing and modeling.

This work's main contribution is as follows:

- We used the ECNN model for the first time to identify blindness symptoms, and we discovered a significant improvement in the recognition of blindness in retinal images, outperforming CNN, and other models with over 92% validation accuracy.
- We utilized a genetic algorithm and a Gaussian filter to help decrease noise and overfitting over the entire image.

Our objective is to automatically identify blind spots in eyes and assess their potential severity using deep learning techniques. In this paper, we propose an enhanced convolutional neural network (ECNN) based on a genetic algorithm. As part of our preprocessing techniques, we employ a Gaussian filter to assist in lowering system noise. The feature extraction procedure has been completed once the preprocessing step has been finished. A genetic algorithm is used to extract the features, which helps to lessen overfitting over the entire image. The classifying stage has now been completed. In our study, a better convolutional neural network is used to improve classification accuracy.

In this paper, Section 2 provides the literature review, Section 3 explained about the overview of CNN approach, Section 4 explains about the proposed work, Section 5 provides the details of dataset, Section 6 shows the results and discussion of the proposed work, and Section 7 concludes the information about this research with future work.

## 2. Review of Literature

Researchers have been focusing on connected smart health, the healthcare Internet of Things (Health IoT), and patient monitoring, all of which offer significant promise for AI and IoT technologies. AI techniques and technologies will improve the situation of world health, particularly for diabetic retinopathy. The numerous challenges and achievements in eye care were discussed by researchers, with a focus on the Indian community. They stated that efficient treatment and potential cures for a number of eye illnesses that may cause vision loss should be available in India by the year 2020. Organizing certification programs for technicians and physicians will help them get the knowledge and abilities necessary for DR identification at an early stage [1–3]. Numerous techniques have been put out to find DR. This section focuses on deep learning and neural network techniques for multiclass classification. Some researchers have classified fundus pictures into two categories: diabetic, which includes moderate to severe NPDR, and nondiabetic, which denotes the person does not have DR [4]. The authors proposed a method for precisely identifying a class into which a fundus picture may be classified based on these results using a single primary classifier and backpropagation neural organizing processes. Similar to this, a deep learning-based method has been developed for classifying fundus images for human ophthalmologist diagnosis. Based on Inception V3, the authors created a Siamese-like CNN binocular model that can give output from both eyes simultaneously and detect fundus images in both eyes [5]. The authors proposed a hybrid approach for DR detection in which the HE contract constrained adaptive histogram supports the deep learning model (CLACHE). The method uses visual augmentation during the diagnosing process to increase attention and effectiveness. For the dataset of individuals with diabetic retinopathy, the authors used five convolutional neural network (CNN) architectures to evaluate progress indicators. According to their classification system, images are split into three groups based on the severity of the disease [6, 7]. Model for diagnosing diabetic retinopathy utilizing convolutional neural networks like AlexNet, VggNet, GoogleNet, and ResNet is put forth. The model uses the classification of the fundus picture of DR into five classes to determine the patient's stage of DR. Additionally, the CNNs model was enhanced for greater accuracy with the use of transfer learning and hyper-parameter tuning, which was not possible when using nontransferred learning for noisy data. Images have been preprocessed using normalization and data augmentation techniques, and noise has been removed using nonlocal means denoising (NLMD) techniques [8–10].

In earlier research, the diabetic retinopathy was identified using a deep learning technology (DR) to capture the discriminative region of the input retina image. The enhanced convolutional neural network (ECNN) layer was examined in the network architecture provided by the authors to identify the total contribution to the final prediction made by the neuron. The authors' proposal distinguishes the precise location of severity in terms of vascular anomalies leads to greater performance.

## 3. Overview of the CNN Approach

**Figure 1:**
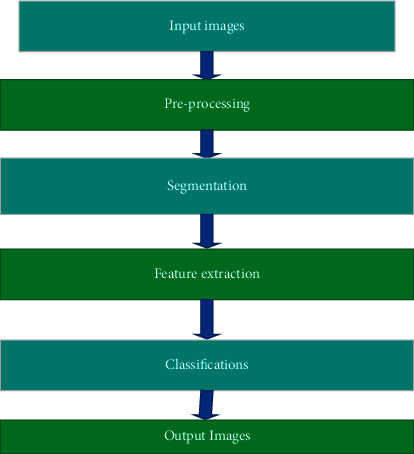
Overview of the proposed approach.

This study implements the image processing applications in the complete network by implementing the recommended approach in its overview for the full network. The preprocessing method, segmentation, feature extraction, and classification process are the fundamental principles of image processing. The output images are then monitored in the form of a view. The proposed approach's implementations are shown in an overview in Figure 1.

The noise removal filter is part of the preprocessing technique, which also improved the image quality. The conversion of RGB photographs to greyscale images is the primary purpose of the preprocessing images. The preprocessing method is regarded as the most important method in image processing.

## 4. Overview of the Proposed Method

In order to address issues in computer vision, deep neural networks built on convolutional neural network models are increasingly commonly used. CNN-based and genetic algorithm approaches were used to distribute the dataset across normal and other types of diabetic retinopathy patients. The adopted CNN models are displayed systematically in the figure below, together with a genetic method for feature selection. By applying filter techniques like Gaussian filtering, it eliminates the input noise in the images. The feature extraction technique is used following the preprocessing procedure.

The quality of the photographs is improved by feature extraction since it helps to divide the pixel images into smaller and easier-to-manage images. A vital part of the image processing system is feature extraction. The construction of good classification accuracy is a result of the higher quality of the feature extraction.

**Figure 2:**
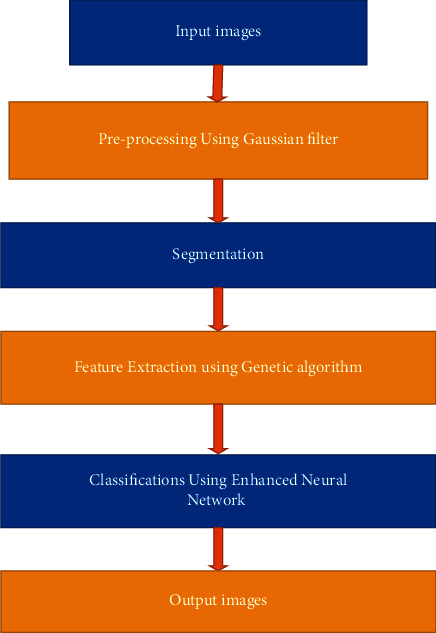
Proposed ECNN.

The proposed enhanced convolutional neural network is implied in Figure 2.

The input images for our suggested method are made up of the datasets that are provided, and the preprocessing operations are conducted using the Gaussian filter. Thus, it aids in the overall image processing process of removing noise. It contributes to the improved image quality. Feature extraction might happen after image processing is finished. A genetic algorithm can be used to handle the feature extraction.

Edge detection and image sharpening can be used to find the genetic algorithm. The overfitting in image processing is lessened as a result. The feature extraction can be finished before using the classification method. The convolutional neural network can be used to handle classification algorithms. It improves outcomes of the high quality of the photos and reduces overfitting in the input photographs.

### 4.1. Preprocessing

The Gaussian filter is a low pass filter that is used in our suggested method to remove noise from the photos. It aids in capturing the completing the image.

In comparison to the current procedures, it thus produces superior boosting effects. The Gaussian filter's key benefit is that it offers filter applications in common models like zero frequency. The use of the Gaussian filter to handle both positive and negative values is demonstrated by this example.

In order to remove blurring and improve image quality, the Gaussian filter is used in image processing. This leads to the feature extraction technique.

(1) $$\begin{array}{c} F\left(x,y\right)=\frac{1}{2*3.14\left(\sigma^{2}\right)}e^{\left(x^{2+y^{2}}/2\sigma^{2}\right)}\text{,} \end{array}$$

where *σ* is the distribution's standard deviation. At this cut-off frequency, the response value of the Gaussian filter is equal to exp (0.5) 0.607. The constant in the last equation that comes before the standard deviation in the frequency domain for *c* = 2 is roughly 1.1774, or half the full width at half maximum (FWHM).

The above equation shows that the sample space functions in the 2D image filtering equations in the Gaussian filter.

### 4.2. Genetic Algorithm

A genetic algorithm (GA) is an optimization technique that has been successfully applied in deep learning research. The GA model is an evolutionary search method that mimics the mechanisms of crossover, mutation, and selection found in nature. Feature selection is the process of selecting the most reliable and discriminating qualities while minimizing the high dimension of the feature space. GA is a metaheuristic feature selection method that starts the search and uncovers a wide range of alternatives. As an optimizer, GA will select the best option from a collection of possibilities.

The quality of the photographs is improved since every pixel inside the images depends on the surrounding pixel range.

The final portion of the session gives the adding sequence in the complete image processing because the genetic algorithm's first step is to anticipate and choose the best spots in the image functions before performing the overfitting functions [21]. The only factor on which the genetic algorithm depends is the system's fitness function.

**Figure 3:**
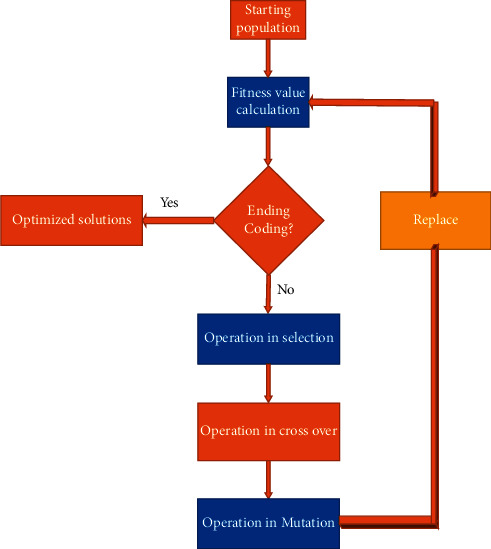
Genetic algorithm.

The genetic approach for extracting eye disease features is shown in Figure 3. The selection function, the crossover function, and the mutation function make up the three main building blocks of the genetic algorithm.

The overfitting functions serve as the foundation for these genetic functions. It aids in the reduction of overfitting image processing processes. The classification approaches use the output results as their input.

### 4.3. Enhanced Convolutional Neural Network

The convolutional neural network provides enhanced picture quality and the main function of the CNN provides the image pixel quality accuracy and also enhanced the classifications of the better quality of inside pixels since it depends on the adjacent pixel.

**Figure 4:**
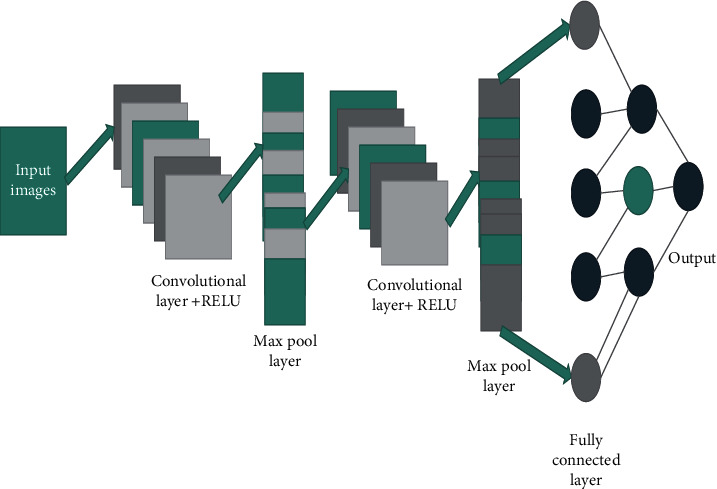
Enhanced convolutional neural network.

Figure 4 represents the enhanced convolutional neural network in image processing.

The first step of the image processing in the classification of the images is to insert the feature extraction input in the entire image processing and then the convoluting layer helps to detect the pixel images, after the completion of the detection of the images and then the Max-pooling technique takes places. Thus, the Max-pooling helps to reduce the overfitting in the image processing.

Sensitivity: A test's sensitivity is how well it can identify the patient cases. We should compute the percentage of true positives in patient situations in order to estimate it. The following can be expressed mathematically as

(2) $$\begin{array}{c} Sensitivity=\frac{TP}{TP+FN}*100\text{.} \end{array}$$

Specificity: A test's specificity is measured by how well it can identify healthy instances. We should compute the proportion of true negative in healthy cases to estimate it. The following can be expressed mathematically as

(3) $$\begin{array}{c} Specificity=\frac{TN}{TN+FP}*100\text{.} \end{array}$$

Accuracy: A test's accuracy is determined by how well it can distinguish between patients and healthy cases. Calculating the proportion of true positive and true negative results in all analyzed cases is necessary to estimate a test's accuracy. The following can be expressed mathematically as

(4) $$\begin{array}{c} Accuracy=\frac{TN+TP}{FN+FP+TN+TP}*100\text{.} \end{array}$$

Indexes: A BTree-index, for example, can be thought of as a model to map a key to the position of a record within a sorted array; a Hash-index, for example, can be thought of as a model to map a key to the position of a record within an unsorted array; and a BitMap-index, for example, can be thought of as a model to indicate whether a data record exists or not.

(5) $$\begin{array}{c} Index=Sensitivity+specificity-1\text{.} \end{array}$$

The above equations show the sensitivity, specificity, accuracy, and index value in the eye diseases classifications.

## 5. Dataset

We have collected 50 sampled images from the public datasets and then the sample images are trained and tested in the public datasets. The preprocessing technique is handled in the Gaussian filter and the feature extraction takes place through the genetic algorithm and the classification techniques can be handled by using the ECNN approach.

## 6. Results and Discussion

Our proposed methods provide enhanced accuracy of the classifications. It gives gradually enhanced outputs when compared to the existing techniques. SVM with the genetic algorithm, convolutional neural networks with the genetic algorithm, and K- closest neighbors with the existing methods are the existing methodologies mentioned in this research. This paper implements enhanced output accuracy when compared to the existing techniques.

**Figure 5:**
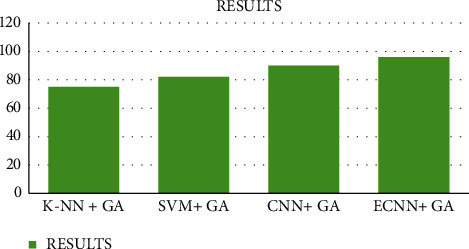
Comparison of accuracy between the proposed work and existing works.

Figure 5 represents the results of our proposed approach compared with the existing approach.

### 6.1. Discussion

Many datasets, like Messidor, IDRiD, and others, include high-quality photographs that were taken under deliberate and unusual circumstances (i.e., similar environmental and hardware conditions across captures). Therefore, it might be argued that algorithms developed using such datasets will struggle in real-world applications where the images may not be same and hardware and ambient factors may vary. On the other hand, even though Kaggle EyePACS and APTOS datasets address these problems and closely resemble a real-world scenario, the noise that results from those variations makes it very challenging for the algorithms to carry out the analysis accurately and effectively because it includes images that were taken from a variety of camera models under various nontypical conditions. However, one can create strong algorithms that can be useful in clinical practice by taking into account those low-quality photos that reflect the actual facts. Poor image quality in the data can have an impact on both the training process and the model's performance. On a low contrast or blurry image, subtle evidence of retinopathy at an early stage can be easily hidden. In their diagnostic process, Tsiknakis et al. [30] introduced a quality evaluation module that eliminates photos from the collection that cannot be graded. These pictures are then sent to a qualified ophthalmologist for analysis. The poor quality photos from the final dataset were likewise disregarded by Paisan Ruamviboonsuk et al. [31]. Definition of the classification as a 6-class scenery grading problem referred to the first 5 classes as the ICDR grading scale procedure and the sixth class as ungradable images, thereby integrating the quality assessment module into deep learning. Prior to clinical integration, two more critical issues relating to the robustness and reliability of the models must be appropriately addressed. The need for the models to continuously function accurately across expected variances found in the clinical environment, including variations about data collected from numerous centers or devices from diverse manufacturers, is finally described by these phrases.

## 7. Conclusion and Future Work

A major side effect of diabetes mellitus, diabetic retinopathy, causes gradual retinal degeneration and can even result in blindness. To stop it from getting worse and harming the retina, it is crucial to find and treat it early. Since numerous DL systems have evolved and been integrated into clinical practice, there has been an increase in interest in using them to diagnose diabetic retinopathy. This will help physicians treat patients more effectively and efficiently. In our paper, the categorization of eye retinal images is implemented using an enhanced convolutional neural network, and feature extraction is performed using a genetic method. As a result, the overfitting issue with image classifications is lessened, and the image quality is also improved. Although deep learning has set the door for more precise diagnosis and therapy, additional advancements in performance, interpretability, and ophthalmologist trustworthiness are still required.

## Data Availability

The datasets used and/or analyzed during the current study are available from the corresponding author on reasonable request.
